# Acute hepatitis in a biological male patient secondary to suspected disseminated gonococcal infection in the absence of Fitz-Hugh–Curtis syndrome: a case report

**DOI:** 10.1186/s13256-025-05709-x

**Published:** 2025-12-29

**Authors:** Robert Levi Snedegar, Jonathan Williamson, Matthew Epperly

**Affiliations:** Department of Family Medicine, West Virginia University, 6040 University Town Center Drive, Morgantown, WV 26501 USA

**Keywords:** Gonococcal infection, Hepatitis, Fitz-Hugh–Curtis syndrome, Case report

## Abstract

**Background:**

Disseminated gonococcal infection is well described in the literature and most commonly presents with signs and symptoms of systemic inflammation including arthritis, tenosynovitis, dermatitis, or imaging findings consistent with Fitz-Hugh–Curtis syndrome. This condition is characterized by inflammation of the liver capsule with adhesion formation—most commonly a sequela of chronic pelvic inflammatory disease. However, there are no documented cases of acute hepatitis secondary to disseminated gonococcal infection in the absence of these findings.

**Case presentation:**

Here we present a case report of a 31-year-old Caucasian biological male presenting with abdominal pain found to have acute hepatitis and *Neisseria* gonorrhoeae bacteremia. He reported no arthritic or dermatologic symptoms, and his imaging findings were not consistent with Fitz-Hugh–Curtis syndrome. His abdominal pain and hepatic injury greatly improved following treatment of his disseminated gonococcal infection with ceftriaxone and doxycycline. Given his remarkable improvement on antibiotic therapy and his otherwise unremarkable workup, his acute hepatitis was strongly suspected to be secondary to disseminated gonococcal infection.

**Conclusion:**

To our knowledge, this is the first reported case of disseminated gonococcal infection causing acute hepatitis in the absence of the previously listed systemic findings or a diagnosis of Fitz-Hugh–Curtis syndrome. This case highlights the diagnostic challenges in disseminated gonococcal infection-related hepatitis and underscores the need to maintain a broad differential diagnosis when managing a patient with acute hepatitis of unknown origin.

**Supplementary Information:**

The online version contains supplementary material available at 10.1186/s13256-025-05709-x.

## Background

Disseminated gonococcal infection (DGI) is a rare but serious complication of *Neisseria gonorrhoeae* infection, often presenting with symptoms such as arthritis, tenosynovitis, and dermatitis [1–5]. In rare cases, DGI can involve visceral organs, leading to complications such as hepatitis or perihepatitis [6]. While Fitz-Hugh–Curtis syndrome, characterized by inflammation of the liver capsule with adhesion formation, has been documented as a potential hepatic manifestation of gonococcal infections, the absence of hepatic capsule distension or liver abscesses has been considered a means of effectively ruling out Fitz-Hugh–Curtis syndrome [7–10].

Acute gonococcal hepatitis in biological males is exceedingly rare, with few cases described in the literature. Most involve either direct liver involvement or secondary complications such as abscess formation or bacteremia [9, 10]. This condition requires careful differentiation from other infectious causes of hepatitis, including adenovirus, which has recently been suspected to cause hepatitis in immunocompromised pediatric patients [11]. Adenovirus can also cause fulminant liver disease in both immunocompromised and immunocompetent adults [12–14]. However, the diagnostic utility of adenovirus serologies is crucial in excluding adenovirus as an etiologic agent. Here, we present a case of DGI causing acute hepatitis in the absence of systemic findings or a diagnosis of Fitz-Hugh–Curtis syndrome.

## Case presentation

A 31-year-old Caucasian biological male with past medical history of syphilis, chlamydia, herpes zoster (all adequately treated), and cholecystectomy presented to the emergency department with 4-day history of epigastric pain after a recent vacation to Key West. The patient reported several sexual contacts during this trip. Upon his return, he had been experiencing a fever of up to 38.5 °C, penile discharge, and diarrhea. Of note, he had been seen in an urgent care clinic each of the two days prior to presentation for the same complaints. Workup at the urgent care had been initiated with a negative polymerase chain reaction (PCR) test for coronavirus disease of 2019 (COVID-19), influenza A, influenza B, and respiratory syncytial virus. Syphilis titer, human immunodeficiency virus screen, *Neisseria* gonorrhoeae RNA, *Chlamydia trachomatis* RNA, and herpes simplex virus type 1 and type 2 screen were still pending at the time of presentation to the emergency department.

In the emergency department, the patient was tachycardic, tachypneic, and febrile. His hepatic function panel was notable for a total bilirubin of 7.8 mg/dL, a conjugated bilirubin of 5.9 mg/dL, an aspartate aminotransferase of 8520 U/L, an alanine aminotransferase of 5814 U/L, and an alkaline phosphatase of 212 U/L. His white blood cell count and hemoglobin were within normal limits. A basic metabolic panel demonstrated hyponatremia with sodium of 131 mmol/L and acute kidney injury with a creatinine level of 1.42 mg/dL from a baseline of 0.9 mg/dL. Venous blood gas was significant for a pH of 7.29 and a lactic acid level of 9.6 mmol/L. His urinalysis demonstrated bilirubin, protein, leukocytes, and white blood cell casts, but urine culture demonstrated no growth. Computed tomography of the chest, abdomen, and pelvis was performed and demonstrated an enlarged steatotic liver and mild stranding around the bladder and appendix, possibly concerning for diverticulitis. He was noted to have a negative hepatitis B and hepatitis C antibody screen. His hepatitis A immunoglobulin G (IgG) was positive, but hepatitis A immunoglobulin M (IgM) was negative, indicating immunity to the virus but with no active infection.

The patient was admitted to the hospital, started on a pain control regimen, and aggressively fluid resuscitated. Owing to his history of high-risk sexual behavior, he was also on emtricitabine, tenofovir, disoproxil fumarate for pre-exposure prophylaxis, and this was held during his admission. Given his degree of hepatic dysfunction, gastroenterology was consulted and recommend hepatitis E IgM, cytomegalovirus, adenovirus, and Epstein–Barr virus serologies. Overnight, his sexually transmitted infection screening from urgent care came back remarkable for a positive *Neisseria* gonorrhoeae and *Chlamydia trachomatis* RNA result. Blood cultures were collected, and he was started on ceftriaxone 1 g daily and doxycycline 100 mg twice daily for treatment of these infections. Magnetic resonance cholangiopancreatography was performed, which confirmed hepatomegaly with a moderate degree of hepatic steatosis, perihepatic ascites, and periportal edema but no evidence of hepatic capsule distention, hepatic capsule adhesions, or hepatic abscesses.

On day 2 of hospitalization, one of his two sets of blood cultures returned positive for *Neisseria* gonorrhoeae. Infectious disease consultation recommended continuing with ceftriaxone and doxycycline and repeating blood cultures. These repeat blood cultures remained negative, and his hepatitis E IgM, cytomegalovirus PCR, Epstein–Barr virus, and adenovirus IgM returned unremarkable. He was noted to have a positive adenovirus IgG. Given his positive blood cultures for *Neisseria* gonorrhoeae and his otherwise unremarkable workup, his acute hepatitis was suspected to be secondary to disseminated gonococcal infection. His liver function tests, lactic acidosis, acute kidney injury, and abdominal pain continued to improve on this antibiotic regimen, and on day 4 of hospitalization he was deemed stable for discharge home with a total of 14 days of ceftriaxone 1 g daily from the date of last negative blood culture and a total of 7 days of doxycycline 100 mg twice per day. The patient followed up as outpatient in the infectious disease clinic, and his liver function tests continued to downtrend. His emtricitabine, tenofovir, disoproxil fumarate was restarted, and he was placed on doxycycline 200 mg for one dose to take as post-exposure prophylaxis within 72 h after a sexual encounter.

After discharge, the patient was seen in clinic at 3 days post discharge with a follow-up hepatic function panel. His aspartate transaminase (AST) and alanine transaminase (ALT) continued to downtrend. A repeat hepatic function panel was collected at 7 days post discharge and 14 days post discharge. At 14 days post discharge, the total bilirubin had normalized at 0.8 mg/dL, conjugated bilirubin was mildly elevated at 0.5 mg/dL, AST normalized at 37 U/L, and ALT normalized at 27 U/L. Another hepatic function panel was not collected until a routine follow-up with his primary care physician at approximately 5 months post discharge, and all components of the hepatic function panel were found to be within normal limits. Tables 1 and 2 provide a timeline of the patient’s course as well as the trend of hepatic function.

**Table 1:**
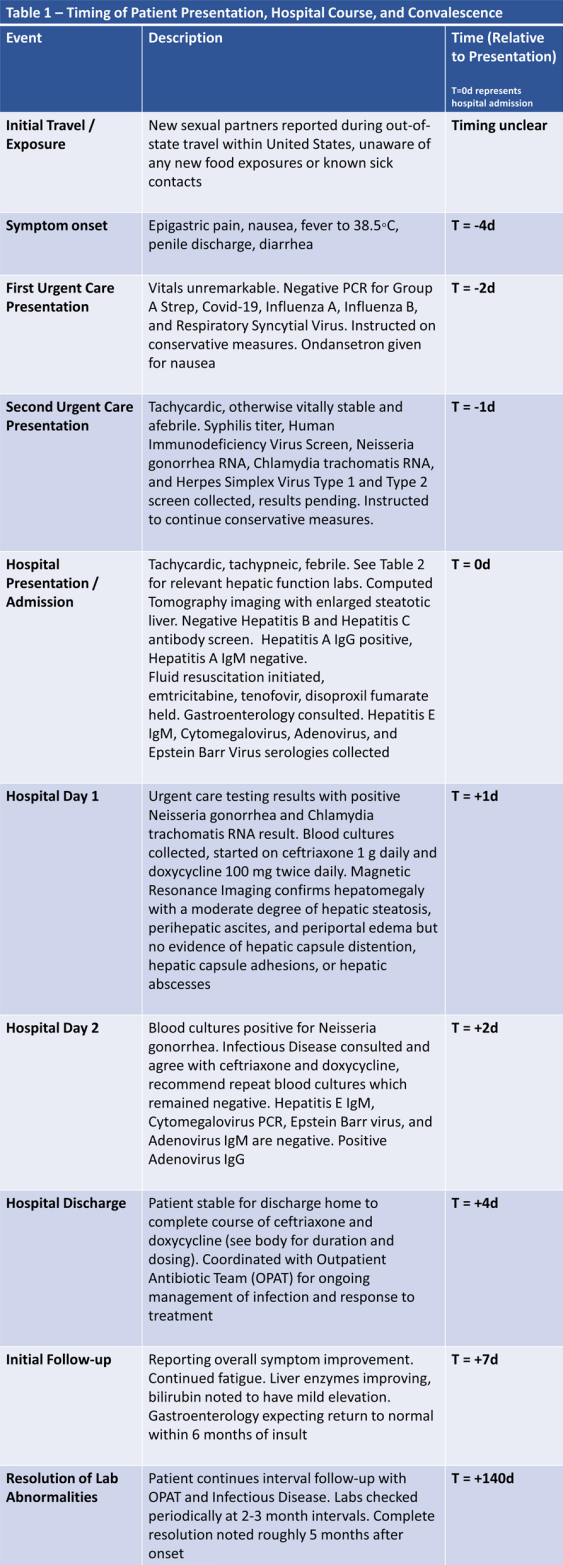
Timing of patient presentation, hospital course, and convalescence

**Table 2:**
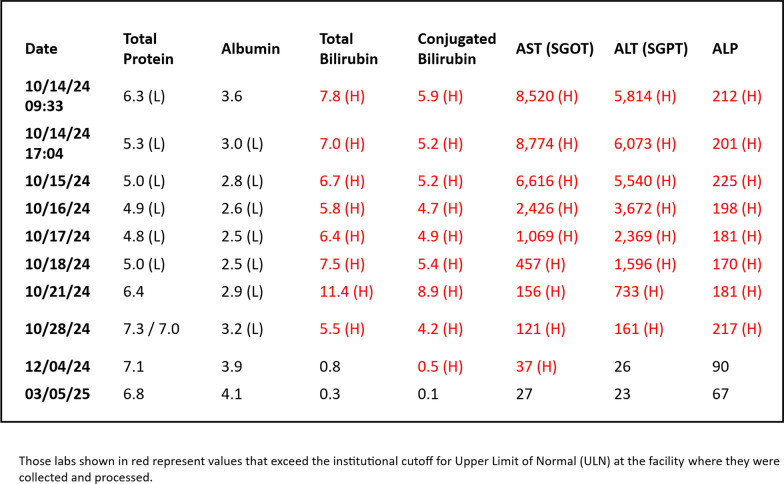
Trend of hepatic function over time

## Discussion and conclusions

This case report presents a unique instance of acute hepatitis in a young, otherwise healthy biological male with DGI, characterized by abnormal liver enzymes and systemic inflammation, but without arthritis, tenosynovitis, dermatitis, or imaging findings consistent with Fitz-Hugh–Curtis syndrome. After review of medical literature indexed in PubMed and Google Scholar, hepatitis in addition to perihepatitis (Fitz-Hugh–Curtis syndrome) and liver abscesses have been reported [9, 10]. However, to our knowledge, this is first reported case of DGI causing acute hepatitis in the absence of the previously listed systemic findings, liver abscesses, or a diagnosis of Fitz-Hugh–Curtis syndrome. While the exact mechanism that causes hepatic injury in this disease process is not fully understood, some have suggested direct invasion of hepatic cells by *Neisseria* gonorrhoeae, ischemia secondary to vascular inflammation, or circulating immune complexes in hepatobiliary infections, which could suggest immune-mediated hepatocellular injury [2, 9, 15]. Regardless of the mechanism, this remains a rather unique case of DGI. A positive adenovirus IgG serology was noted but was deemed an unlikely contributor owing to the absence of IgM reactivity. This report highlights the diagnostic challenges in DGI-related hepatitis and underscores the importance of maintaining a broad differential diagnosis when managing a patient with acute hepatitis of unknown origin, including DGI even in the absence of classic symptoms.

## Supplementary Information

Additional file1 (PDF 3453 kb)

## Data Availability

All applicable data reported in this manuscript are housed in our electronic health record.

## Abbreviation

DGI: Disseminated gonococcal infection
