# Supplementary material for: Acute hepatitis in a biological male patient secondary to suspected disseminated gonococcal infection in the absence of Fitz-Hugh–Curtis syndrome: a case report
Source: J Med Case Rep. 2025 Dec 29;19:653. doi: 10.1186/s13256-025-05709-x (PMC12751404; doi:10.1186/s13256-025-05709-x)
Supplement: Supplementary file 1 — Additional file1 (PDF 3453 kb) [file 13256_2025_5709_MOESM1_ESM.pdf]

CARE
case report guidelines

CARE Checklist of information to include when writing a case report

(cc) BY-NC-ND

✓

Topic	Item	Checklist item description	Reported on Line
Title	1	The diagnosis or intervention of primary focus followed by the words "case report"	Title Page, 8
Key Words	2	2 to 5 key words that identify diagnoses or interventions in this case report, including "case report"	20
Abstract (no references)	3a	Introduction: What is unique about this case and what does it add to the scientific literature?	30-32
	3b	Main symptoms and/or important clinical findings	42-62
	3c	The main diagnoses, therapeutic interventions, and outcomes	79-81, 81-88
	3d	Conclusion—What is the main "take-away" lesson(s) from this case?	95-98
Introduction	4	One or two paragraphs summarizing why this case is unique (**may include references**)	95-97
Patient Information	5a	De-identified patient specific information.	40-42
	5b	Primary concerns and symptoms of the patient.	41-45
	5c	Medical, family, and psycho-social history including relevant genetic information	40-42
	5d	Relevant past interventions with outcomes	40-42
Clinical Findings	6	Describe significant physical examination (PE) and important clinical findings.	50-62
Timeline	7	Historical and current information from this episode of care organized as a timeline	40-45
Diagnostic Assessment	8a	Diagnostic testing (such as PE, laboratory testing, imaging, surveys).	50-62
	8b	Diagnostic challenges (such as access to testing, financial, or cultural)	79-81
	8c	Diagnosis (including other diagnoses considered)	79-81
	8d	Prognosis (such as staging in oncology) where applicable	N/a
Therapeutic Intervention	9a	Types of therapeutic intervention (such as pharmacologic, surgical, preventive, self-care)	81-85
	9b	Administration of therapeutic intervention (such as dosage, strength, duration)	81-85
	9c	Changes in therapeutic intervention (with rationale)	N/a
Follow-up and Outcomes	10a	Clinician and patient-assessed outcomes (if available)	81-88
	10b	Important follow-up diagnostic and other test results	81-88
	10c	Intervention adherence and tolerability (How was this assessed?)	81-88
	10d	Adverse and unanticipated events	N/a
Discussion	11a	A scientific discussion of the strengths AND limitations associated with this case report	79-81
	11b	Discussion of the relevant medical literature **with references**.	22-37
	11c	The scientific rationale for any conclusions (including assessment of possible causes)	79-81
	11d	The primary "take-away" lessons of this case report (without references) in a one paragraph conclusion	91-100
Patient Perspective	12	The patient should share their perspective in one to two paragraphs on the treatment(s) they received	N/a
Informed Consent	13	Did the patient give informed consent? Please provide if requested	Yes ☒ No ☐
